# The Origin of Vertebrate Gills

**DOI:** 10.1016/j.cub.2017.01.022

**Published:** 2017-03-06

**Authors:** J. Andrew Gillis, Olivia R.A. Tidswell

**Affiliations:** 1 Department of Zoology, University of Cambridge, Downing Street, Cambridge, CB2 3EJ, UK; 2 Marine Biological Laboratory, 7 MBL Street, Woods Hole, MA 02543, USA

**Keywords:** gills, chondrichthyan, skate, evolution, vertebrate, pharyngeal arch, endoderm, development, homology

## Abstract

Pharyngeal gills are a fundamental feature of the vertebrate body plan [1]. However, the evolutionary history of vertebrate gills has been the subject of a long-standing controversy [2, 3, 4, 5, 6, 7, 8]. It is thought that gills evolved independently in cyclostomes (jawless vertebrates—lampreys and hagfish) and gnathostomes (jawed vertebrates—cartilaginous and bony fishes), based on their distinct embryonic origins: the gills of cyclostomes derive from endoderm [9, 10, 11, 12], while gnathostome gills were classically thought to derive from ectoderm [10, 13]. Here, we demonstrate by cell lineage tracing that the gills of a cartilaginous fish, the little skate (*Leucoraja erinacea*), are in fact endodermally derived. This finding supports the homology of gills in cyclostomes and gnathostomes, and a single origin of pharyngeal gills prior to the divergence of these two ancient vertebrate lineages.

## Highlights

• The gills of cartilaginous fishes derive from endoderm
• Gills in cyclostomes and gnathostomes primitively shared a common embryonic origin
• Pharyngeal gills are a shared, primitive feature of the vertebrate crown group

## Teaser

Gillis and Tidswell use cell lineage tracing in embryos of the little skate (*Leucoraja erinacea*) to demonstrate the endodermal origin of gills. This finding supports the homology of cyclostome and gnathostome gills and overturns the hypothesis that gills evolved independently in these two ancient vertebrate lineages.

## Results and Discussion

**Figure 1:**
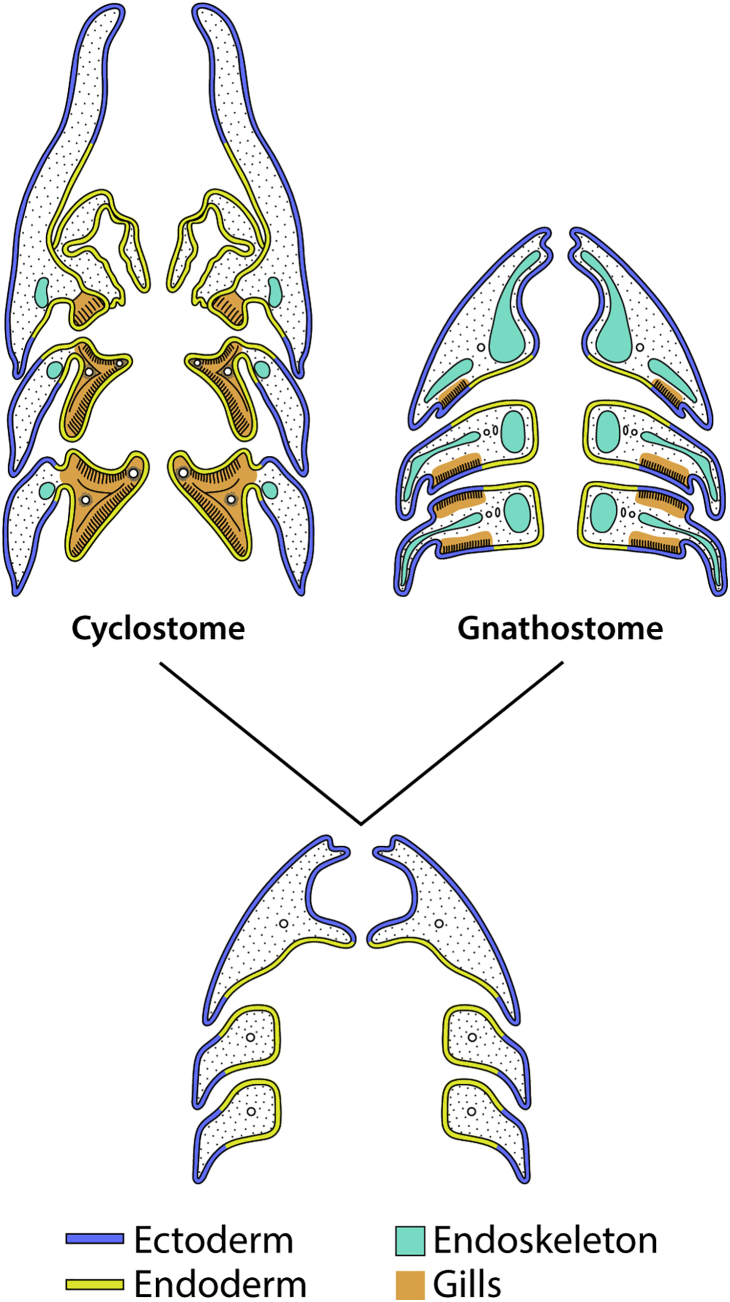
The Ecto-endobranchiate Hypothesis The independent evolution of gills in cyclostomes and gnathostomes (from a gill-less common ancestor), based on their distinct embryonic origins from endoderm and ectoderm, respectively. Redrawn after Jarvik [3] and Jollie [5].

Pharyngeal gills are a defining feature of vertebrate animals and are present as vestiges in our own embryology. However, it has been proposed that the gills of cyclostomes and gnathostomes evolved independently. This “ecto-endobranchiate hypothesis” (Figure 1) extends from the observation that the gills of cyclostomes and gnathostomes form on different regions of the branchial arches (medial versus lateral to the gill endoskeleton, respectively) and arise from distinct embryonic epithelia (endodermal versus ectodermal, respectively) [2, 3, 4, 5, 6, 7, 8]. While the topological incongruence of gills and their skeletal support is not generally regarded as an insurmountable obstacle to homology [7, 8, 14], the issue of distinct embryonic origins is potentially more problematic and remains unresolved.

**Figure 2:**
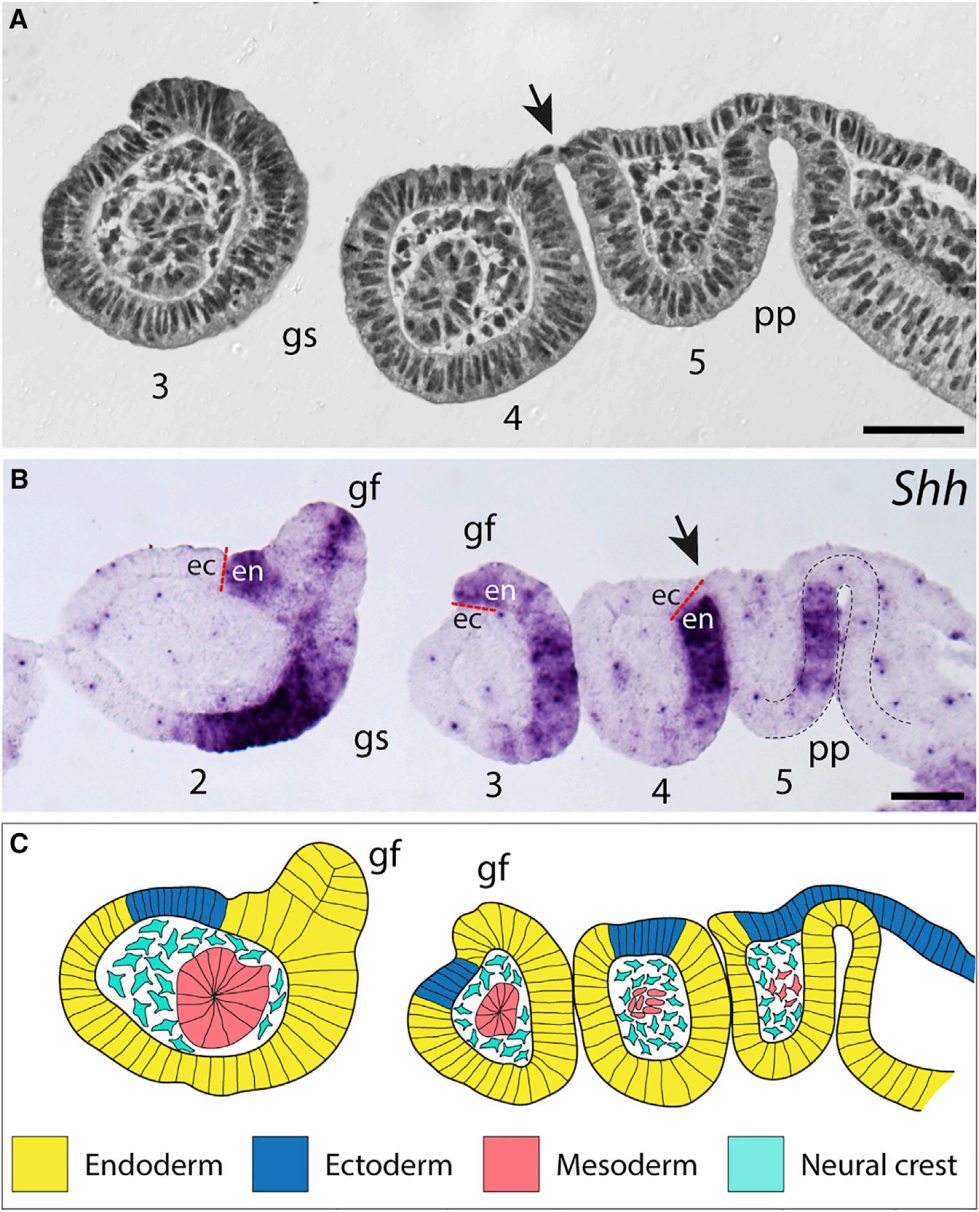
Skate Gill Filaments Arise within an Endodermal *Shh* Expression Domain (A) At stage 22, the sequence of pharyngeal arch formation may be captured along the rostro-caudal axis of a single embryo (rostral to the left). Endodermally derived pharyngeal pouches (pp) contact surface ectoderm and ultimately fuse with this ectoderm (black arrow), giving rise to a gill slit (gs). The columns of tissue that are isolated by adjacent gill slits are pharyngeal arches (pharyngeal arches 3, 4, and 5 shown here). (B) *Shh* is expressed along the anterior wall of each pharyngeal pouch (pp) and, eventually, along the posterior wall of each pharyngeal arch (pharyngeal arches 2, 3, 4, and 5 shown here). The red dashed line at the interface between *Shh*-expressing and non-expressing epithelia indicates the predicted interface between endoderm (en) and ectoderm (ec). Note that early gill filaments (gf) arise within *Shh*-expressing epithelium. The black dashed lines delineate caudal pharyngeal pouch endoderm, and the black arrow indicates a pharyngeal pouch fusing with overlaying surface ectoderm. (C) Schematic illustrating predicted tissue contributions to skate pharyngeal arches. Based on histological and gene expression analyses, we predict that gill filaments (gf) derive from endodermal epithelium. Scale bars represent 40 μm.

In fishes, gills develop on pharyngeal arches, paired columns of tissue that are bound by ectodermal and endodermal epithelia and form from the walls of the embryonic foregut [15]. Pharyngeal arch development begins with the iterative outpocketing of foregut endoderm in a rostral-to-caudal sequence, giving rise to a series of endodermal pouches [16]. These pouches subsequently contact and fuse with overlying surface ectoderm, resulting in the perforation of gill slits and the delineation of arches (Figure 2A). The endodermal origin of gills in lamprey and hagfish is well established from classical histological studies [9–12], but the putative ectodermal origin of gills in bony fishes is less well documented. Goette described the early development of gills in sturgeon and noted that gill filaments arise from pharyngeal ectoderm, appearing first on the outside of the pharynx, prior to perforation of the gill slits [10]. Kellicott also proposed an ectodermal origin of the gills in lungfish, noting an inward migration of ectodermal epithelium following gill slit perforation but prior to gill filament differentiation [13]. However, cell lineage tracing experiments have demonstrated an endodermal origin of gills in zebrafish [17]. Thus, it remains unclear whether the gills of bony fishes primitively developed from endoderm or ectoderm. We therefore sought to investigate the embryonic origin of the gills in a cartilaginous fish, the little skate, *Leucoraja erinacea*—an outgroup to the bony fishes, which may permit the inference of primitive anatomical and developmental conditions in the last common ancestor of jawed vertebrates.

In the stage 22 (S22) skate embryo [18], the gene encoding the developmental signaling molecule Sonic hedgehog (Shh) is expressed in the anterior endodermal domain of each developing pharyngeal pouch. Once the pouches have fused with the overlying ectoderm, this *Shh* expression domain comprises the posterior epithelium of each pharyngeal arch, where it functions to establish the anterior-posterior axis of the arch [19]. We have noted that the first gill buds, which differentiate from pharyngeal arch epithelium shortly after gill slit perforation, form within this *Shh* expression domain (Figure 2B). Given the endodermal origin of this *Shh* expression domain, this suggests that these gill buds are endodermally derived (Figure 2C), though we cannot rule out that the pharyngeal *Shh* expression domain expands to include adjacent pharyngeal ectoderm upon gill slit perforation.

**Figure 3:**
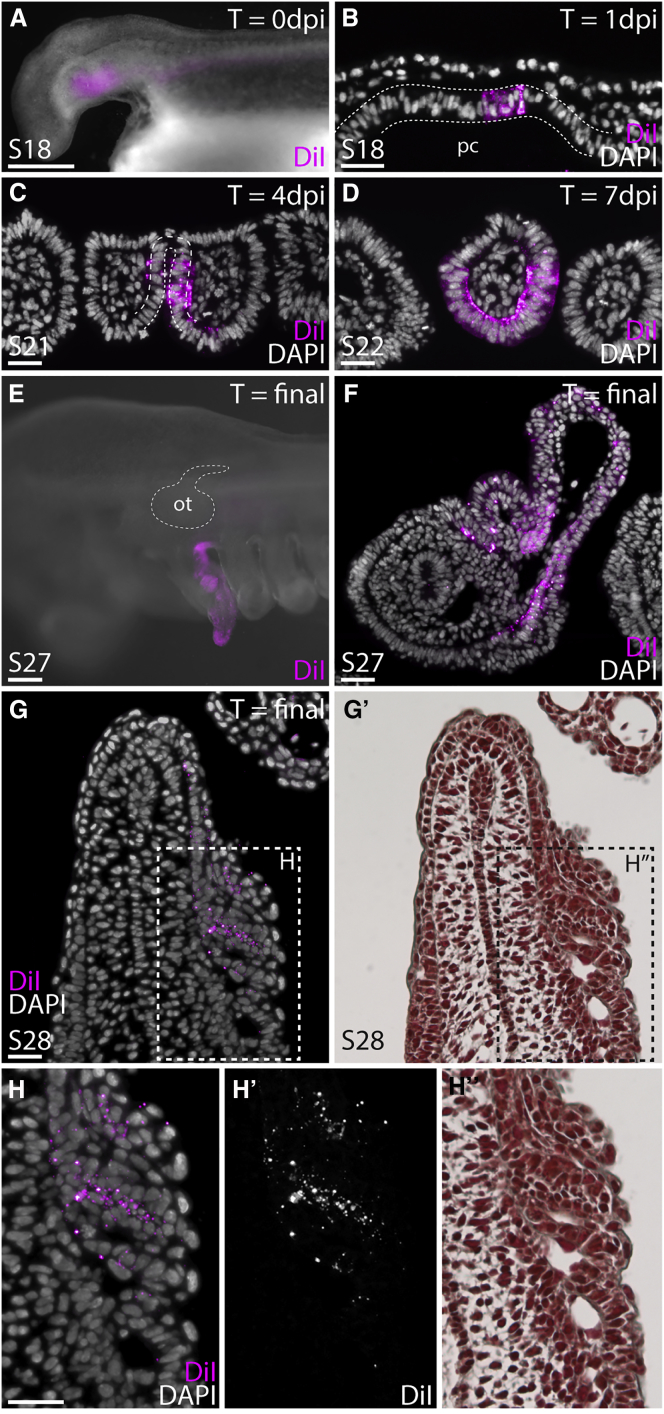
Skate Gills Derive from Pharyngeal Endoderm (A and B) Microinjection of the pharyngeal cavity (pc) of skate embryos with CM-DiI at stage 18 (A) allows us to focally label cells (B) within the pharyngeal endoderm. (C and D) CM-DiI-labeling of endodermal epithelium allows visualization of endodermal contributions to the pharyngeal arches prior to (C) and immediately following (D) the perforation of pharyngeal pouches with overlying ectoderm. Endodermally derived epithelium encircles approximately 3/4 of the circumference of the pharyngeal arches. (E and F) By stage 27, CM-DiI-labeled external gill filaments can be observed in whole-mount (E) and in histological section (F), indicating their endodermal origin. (G and G′) By stage 28, CM-DiI-positive internal gill filaments are also observed in histological section, indicating their endodermal origin. Image in (G′) is the same section as in (G), stained with hematoxylin and eosin. (H–H″) Gills of a stage 28 skate embryo (dashed inset box in G and G′), showing CM-DiI-positive gill filaments. Images in (H′) and (H″) are the same section as in (H), stained with CM-DiI only (H′) and hematoxylin and eosin (H″).

To directly test the embryonic origin of the gills in skate, we conducted a series of pharyngeal endodermal fate mapping experiments. We microinjected the lipophilic dye CM-DiI into the pharyngeal cavity of skate embryos at S18 (prior to the perforation of gill slits) (Figure 3A), and histological examination of labeled embryos 1 day post injection (dpi) (n = 3) revealed that this strategy allows us to focally label regions of pharyngeal endoderm without contaminating overlying ectoderm (Figure 3B). An analysis of labeled embryos at S22 (∼4–7 dpi; n = 3) allowed us to visualize the endodermal contributions to the pharyngeal arches immediately prior to (Figure 3C) and following (Figure 3D) gill slit perforation. Upon their initial delineation by adjacent gill slits, approximately 3/4 of the epithelium surrounding a pharyngeal arch derives from endoderm, with only the lateralmost 1/4 deriving from ectoderm. By S27/28, differentiated gill filaments are present on the hyoid and gill arches. Elasmobranch embryos possess both internal and transient external gill filaments, with the latter ultimately resorbing and remodeling into internal filaments [20]. In labeled embryos reared to S27/28 (∼40–50 dpi; n = 7), CM-DiI-positive external gill filaments were readily apparent (Figure 3E), while histological analyses revealed CM-DiI-positive epithelium on both the external (Figure 3F) and internal gill filaments (Figures 3G and 3H). These experiments, which allow us to directly trace derivatives of pharyngeal endodermal epithelium, demonstrate the endodermal origin of gills in the little skate.

### Conclusions

While the evolutionary origin of pharyngeal arches has been resolved to the deuterostome stem [21, 22, 23], the evolutionary history of gills derived from pharyngeal arch epithelia remains contentious. Gill structures are preserved in the stem vertebrates *Myllogkunmingia* [24], *Metaspriggina* [25], and *Haikouichthys* [26], but in the absence of consensus regarding the homology of gills within the crown group, it has remained unclear to what extent these structures may inform the nature of gills in the last common ancestor of vertebrates. Our demonstration of an endodermal origin of gills in a cartilaginous fish bolsters the homology of gills in cyclostomes and gnathostomes, and the single origin of pharyngeal gills prior to the divergence of these two ancient vertebrate lineages. Our findings, along with recent phylogenetic [27] and paleontological [25] advances, contribute to an emerging picture in which the crown ancestor of vertebrates was more complex—and exhibited more gnathostome-like anatomical conditions—than was previously appreciated, and are consistent with scenarios in which gills evolved along the vertebrate stem, in conjunction with a more active lifestyle [1].

## Experimental Procedures

### Embryo Collection

*L. erinacea* eggs were obtained at the Marine Biological Laboratory (Woods Hole, Massachusetts, USA) and maintained in a flow-through seawater system at ∼17°C to the desired developmental stage. Embryos were fixed in 4% paraformaldehyde in PBS overnight at 4°C, rinsed three times in PBS, dehydrated into 100% methanol, and stored at −20°C. All animal work complied with protocols approved by the Institutional Animal Care and Use Committee at the Marine Biological Laboratory.

### Histology and mRNA In Situ Hybridization

For histological analysis, embryos were embedded in paraffin wax and sectioned at 7 μm as previously described [28]. Sections of CM-DiI-labeled embryos were rehydrated and coverslipped with Fluoromount G containing DAPI (Southern Biotech). Sections were subsequently decoverslipped in water and stained with hematoxylin and eosin. In situ hybridization experiments for *L. erinacea Shh* (GenBank: EF100667) were performed on paraffin sections as previously described [28].

### Fate Mapping

For CM-DiI fate mapping experiments, eggs containing embryos at S18 were windowed, and CellTracker CM-DiI (ThermoFisher Scientific; 0.5 μg/μL, prepared by diluting a 5 μg/μL ethanol stock 1:10 in 0.3M sucrose) was microinjected into the pharyngeal cavity using a pulled glass capillary needle and a Picospritzer pressure injector. Eggs were then sealed with a piece of donor eggshell and Krazy Glue and were left to develop in a flow-through seawater table for 1–50 days prior to fixation.

## Author Contributions

J.A.G. conceived the study and conducted and analyzed the cell lineage tracing experiments. J.A.G. and O.R.A.T. conducted and analyzed the mRNA in situ hybridization experiments. J.A.G. wrote the manuscript and prepared the figures with input from O.R.A.T.
